# A mathematical model for pancreatic cancer during intraepithelial neoplasia

**DOI:** 10.1098/rsos.240702

**Published:** 2024-10-30

**Authors:** Joshua Briones-Andrade, Guillermo Ramírez-Santiago, J. Roberto Romero-Arias

**Affiliations:** ^1^ Facultad de Ciencias, Universidad Nacional Autónoma de México, Ciudad de Mexico, Mexico; ^2^ Instituto de Matemáticas, Universidad Nacional Autónoma de México, Juriquilla, Querétaro, Mexico; ^3^ Instituto de Investigaciones en Matemáticas Aplicadas y en Sistemas, Universidad Nacional Autónoma de México, Ciudad de Mexico, Mexico

**Keywords:** network regulation, negative and positive feedback, delay differential equations, mechanical forces, inflammation

## Abstract

Cancer is the result of complex interactions of intrinsic and extrinsic cell processes, which promote sustained proliferation, resistance to apoptosis, reprogramming and reorganization. The evolution of any type of cancer emerges from the role of the microenvironmental conditions and their impact of some molecular complexes on certain signalling pathways. The understanding of the early onset of cancer requires a multiscale analysis of the cellular microenvironment. In this paper, we analyse a qualitative multiscale model of pancreatic adenocarcinoma by modelling the cellular microenvironment through elastic cell interactions and their intercellular communication mechanisms, such as growth factors and cytokines. We focus on the low-grade dysplasia (PanIN 1) and moderate dysplasia (PanIN 2) stages of pancreatic adenocarcinoma. To this end, we propose a gene-regulatory network associated with the processes of proliferation and apoptosis of pancreatic cells and its kinetics in terms of delayed differential equations to mimic cell development. Likewise, we couple the cell cycle with the spatial distribution of cells and the transport of growth factors to show that the adenocarcinoma evolution is triggered by inflammatory processes. We show that the oncogene RAS may be an important target for developing anti-inflammatory strategies that limit the emergence of more aggressive adenocarcinomas.

## 1. Introduction

Pancreatic ductal adenocarcinoma is among the seventh leading causes of cancer-related deaths worldwide due to its extreme difficulty to be detected and treated [1,2]. There are different types of pancreatic cancer, but the most common is adenocarcinoma in about 95% of cases. It originates in exocrine cells in the pancreatic duct lining. Because the pancreas is located deep in the body, a pancreatic tumour is impossible to detect during a standard physician’s examination. What complicates the situation even more is the fact that there are no typical symptoms of a pancreatic tumour until metastasis occurs. To better understand the key molecular mechanisms of the development of pancreatic cancer, researchers have recently focused on analysing the role of microenvironment. The microenvironment consists of diverse components that include metabolites, fibroblasts, endothelial cells, immune cells, as well as endocrine cells, that interact with each other together with cancer cells in a complex fashion.

Unlike normal cells, tumour cells often increase their glucose consumption and lactate production even in the presence of physiological oxygen concentration values and functional mitochondria. The first step of cellular glucose consumption is mediated by solute carriers of the glucose transporter (GLUT) family. The upregulation of GLUTs has been reported in numerous cancer types as a result of perturbation of gene expression or protein relocalization and/or stabilization [3]. There are 14 GLUT proteins that are classified into three classes according to their sequence similarity, namely Class 1 (GLUTs 1–4 and 14), Class 2 (GLUTs 5, 7, 9 and 11) and Class 3 (GLUTs 6, 8, 10, 12 and 13/HMIT) [3]. Recent investigations have identified GLUT1 and GLUT3 as the main proteins that accelerate cell metabolism [4]. High expression of GLUT1 and/or GLUT3 has been associated with poor patient survival in colorectal carcinoma, breast carcinoma, lung adenocarcinoma, squamous cell carcinoma, ovarian carcinoma and glioblastoma [5–10]. As discussed in [3], deregulated GLUT1 and GLUT3 expression happens either via direct interaction with gene-regulatory elements or by controlling the cellular trafficking of the membrane transporters.

The microenvironment can be modelled with a multiscale model which is based on biological regulation that consists of complex dynamical interacting biomolecular networks. Transcription factors control the production of other transcription factors and kinases as well as the activation of other kinases and cell behaviour. Multiscale modelling of pancreatic carcinoma considers the role of pancreatic cancer cells (PCCs) and pancreatic stellate cells (PSCs), cytokines and growth factors, which are responsible for intercellular communication between the PCCs and PSCs. Major contributors to this microenvironment include immune cells, endothelial cells, nerve cells, lymphocytes, dendritic cells, the extracellular matrix and stellate cells. It has been found that molecules and cells surrounding the PCCs significantly impact the cancer’s response to therapy. Recent studies have also shown that cancer cells secrete numerous types of cytokines, which play an important role in intracellular communication between PCCs and PSCs [11–13].

Populations of cells, multiple time scales and diverse size scales, as well as complex nonlinear dynamics in the response of gene-regulatory networks (GRNs), usually yield fixed points, attractors and different phenotypes. Even though a multiscale model would provide more realistic results, one lacks available control methods that can be applied to the analysis of these models. In spite of this, one can build up a Boolean network (BN) approximation of the model to get a kind of calibration or a guide to the analysis of a continuous multiscale model [14]. With the BN approximation, one can carry out a discrete dynamical analysis in search of phenotype targets, fixed points and attractors as well as GRN responses to different inputs. In addition, network motifs are important substructures of biological GRNs that describe negative feedback, feed-forward regulation and cascades [15–17]. The functioning of these network motifs often depends on emergent properties of many different parameters, especially time delays [18–21]. Delays are crucial to the behaviour of genetic oscillators as well as in development and disease [16,18,22,23]. They can lead to significant biological changes, for instance, sustained oscillations [19,20,22,23]. The aim of this work is to relate cell phenotypes associated with genetic changes in pancreatic ductal adenocarcinoma (PDAC) with the dynamics of time-delayed differential equations.

PDAC is believed to form in the epithelial cell layer of the gland tissue [24]. It originates in a step-wise progression from healthy epithelium to precursor lesion to invasive carcinoma. It is believed to arise from a combination of acute trauma, chronic inflammation and spontaneous genetic mutations of oncogenic *KRAS* and tumour suppressor gene (TSG) [25–27]. *KRAS* activating mutations found in humans impair intrinsic GTPase activity of the KRAS protein and can block the interaction between *KRAS* and GAPs. This leads to constitutive activation of *KRAS* and persistent stimulation of downstream signalling pathways that drive many of the cancer hallmarks such as sustained proliferation, metabolic reprogramming, anti-apoptosis, remodelling of the tumour microenvironment, evasion of the immune response, cell migration and metastasis [27,28]. Specifically, oncogenic *KRAS* reprogrammes PDAC cells to a highly anti-apoptotic state [29].

Resistance to apoptosis makes PDAC highly resistant to the mitochondrial pattern of apoptosis-regulated cell death. Here, it is important to note that *KRAS*, together with *HRAS* and *NRAS* expressed genes belong to the RAS family of proteins that are proto-oncogenes and are frequently mutated in human cancers. These expressed genes are GTPases that function as molecular switches regulating pathways responsible for proliferation and cell survival [30].

**Figure 1.**
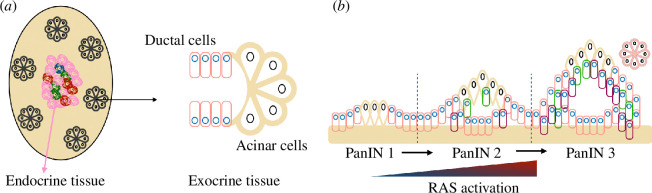
Typical pancreatic tissues. (*a*) Depending on their cell of origin, there are different types of pancreatic cancers. The pancreas can be divided into two tissue types: endocrine and exocrine. Endocrine tissue contains hormone-producing islet cells while exocrine tissue contains enzyme-producing glands. In exocrine glands, acinar cells lead to non-ductal neoplasms whereas ductal cells give rise to ductal neoplasms. (*b*) Pancreatic intraepithelial neoplasias (PanINs) and their transitions with RAS gene activation.

There have been found four distinct precursor lesions that vary in their degree of dysplasia and propensity to develop into infiltrating carcinoma [31]: intra-ductal papillary mucinous neoplasms (IPMNs), mucinous cystic neoplasms (MCNs), intraductal tubulo-papillary neoplasms (ITPNs) and pancreatic intraepithelial neoplasms (PanINs) [31]. IPMNs, MCNs and ITPNs are visible neoplasias that originate in the ducts’ epithelial cell layer and invade the lumen and surrounding connective tissue. PanINs are microscopic non-invasive lesions ( ⪅5 mm) that are located well inside the small intralobular pancreatic ducts. They constitute the most common precursor lesion and cannot be detected or surgically resected as in the other precursor lesions [32]. PanINs are sub-classified into PanIN 1, PanIN 2 and PanIN 3 lesions according to the degree of architectural changes and cytological abnormalities [33]. PanINs can evolve starting at grade 1 (PanIN 1) and progress through PanIN 2 and PanIN 3 to malignancy [34]. In figure 1, we show a typical pancreatic tissue. The risk of these lesions forming invasive carcinoma depends on their genetic stability, location and size inside the pancreas. However, it is strongly believed that most PDAC cases originate from PanIN lesions in cells having developed oncogenic KRAS mutations [33,35]. Taking this into account, here we present and analyse a multiscale qualitative model that mimics the evolution of low-grade dysplasia (PanIN 1) and moderate dysplasia (PanIN 2) stages of pancreatic adenocarcinoma.

## 2. Methods

The pancreas is a complex system made up of several separate functional units that regulate two main physiological processes: digestion and glucose metabolism [36]. In this study, we will focus on the most common pancreatic cancer that involves the exocrine pancreas which is made up of acinar and ductal cells. It originates from the exocrine cells of the lining of the pancreatic duct (see [37] and references therein and [38,39]). The acinar cells that make up the majority of the tissue are arranged in grape-like clusters found at the smaller ends of the branching duct system.

The ducts, which supply an enzyme mixture, in turn, form a network of increasing size that culminates in the main pancreatic ducts. The symmetry of the three-dimensional structure of both the pancreas ductal and acinar cells allows us to model it as a two-dimensional projection that represents the end of the duct and the beginning of the acinar group cells in the form of a ‘U’. This approximation simplifies the numerical analysis of the model in two-dimensional space. One can compare the two-dimensional patterns obtained from the simulations with histological or optical observations from clinical trials.

PanINs are microscopic neoplastic lesions in smaller pancreatic ducts and are often associated with pancreas lobule centric atrophy. PanIN 1 is flat or papillary with columnar epithelium and basally oriented, round nuclei. PanIN 2 is papillary with nuclear hyperchromasia, crowding and pseudostratification. PanIN 3 is papillary, micropapillary or cribriform with nuclear pleomorphism, frequent loss of nuclear orientation and mitosis [40]. During the stages of neoplasia evolution, cell proliferation rates increase, and morphological alterations are increasingly divergent in relation to normal channels, suggesting the generation of abnormal cells in their growth.

The identification of common mutational profiles in simultaneous lesions has provided evidence of the relationship between PanINs and the pathogenesis of pancreatic adenocarcinoma [36]. Specifically, common mutation patterns in PanINs and adenocarcinoma associated with the *RAS* oncogene family have been correlated. Thus, activating mutations on the *RAS* family genes can lead to a remarkable variety of cellular effects, including the induction of proliferation, survival and invasion through stimulation of various signalling pathways, including the regenerative capacity of cells and the multipotent differentiation capacity. As a consequence, acinar cells can transdifferentiate into ductal cells in the absence of cell division and newly emerging ductal cells can subsequently redifferentiate into stem cells.

Some *in vitro* studies [36,41] suggest that identifying the genetic alterations of the *KRAS* gene is not enough to understand the development of neoplasia. It is also necessary to consider the processes of inflammation or stress conditions and tissue damage as well as generalized activation of *KRAS* [38,39,42,43]. In this sense, it has been shown that the induction of an inflammatory process generates different predispositions of exocrine cells to tumourigenesis. In addition, it has been shown that acinar and ductal cell lesions may affect the probability of tumour formation.

### 2.1. Model

Based on the shape and spatial arrangement of the pancreas, one can conclude that cell reproduction in the early stages of pancreatic cancer development mainly involves three intertwined dynamics, namely cell proliferation, inflammatory process and glucose concentration gradients. The occurrence of glucose concentration gradients provides the spatial information necessary to regulate each cell proliferation rate and ultimately determine its phenotype. Considering that the elastic properties of the tissues are being used to assist in the diagnosis of solid pancreatic lesions (SPLs) [44–48], we propose three hypotheses to model pancreatic lesions and pancreatic intraepithelial neoplasms.

Our first hypothesis is that the conditions imposed by the extracellular matrix regulate the processes of growth and proliferation. It has been found that in both mice and humans, tissue stiffness increases in proportion to tumour size, indicating that variation of the mechanical stiffness is an ongoing process during tumour progression [44]. For simplicity, here we propose that there exists a potential energy associated with the elastic mechanical field in the ductal and acinar tissues that can be modelled with the second-order polynomial in the tissue deformation [49]. With this characterization, tissue’s local and global elastic properties can be described without difficulty. We need specific functions of the elastic stress, mechanical pressure as well as other local mechanical forces.

Our second hypothesis considers that cell proliferation and growth require the consumption of glucose. Because the transport of glucose depends on the tissue stiffness, one can assume that spatio-temporal patterns of glucose concentration in the acinar and ductal tissue are shaped by the local elastic field [14,50] and elastic tissue relaxation [51–54].

Our third hypothesis assumes that at places where cells divide and tissue expands the elastic field changes which produce glucose concentration gradients that determine the local cell proliferation rate.

According to these hypotheses, we model the elastic properties of the pancreas ductal and acinar cells by means of a potential function that depends on time and space. The negative gradient of this function yields the mechanical force. Taking advantage of the spatial symmetry of the acinar cells, the tissue is represented by a two-dimensional tessellation that leads to a set of polygonal cells covering the space without any gaps or overlaps. To this end, we use Voronoi diagrams obtained from a collection of scattered random points on a Euclidean plane. Each point represents the position of each biological cell. In the electronic supplementary material, Voronoi diagrams section, a detailed construction of Voronoi diagrams is presented as well as ts relation with the mechanical forces and glucose transport.

### 2.2. Gene-regulatory network

Different methodologies and approaches have been developed to model GRN dynamic interactions. For instance, aspects such as cooperation, competition and regulation have been analysed [37,55,56]. Analogously, for the models’ mathematical formulation and analysis, aspects such as simulation of logic gates [57], network motifs [16], design of genetic circuits [58] and modelling of genetic circuits [59] have also been considered. (See the electronic supplementary material, Gene regulatory network section, for details of the modified pancreatic GRN.)

Models based on sets of ordinary differential equations (ODEs) have been proposed to describe complex biological phenomena, such as population growth, cell signalling pathways, cell-to-cell communication, enzyme kinetics, among others [60,61]. Despite of being formed by relatively simple systems of ODEs, these models are able to capture many nonlinear features of the biological systems.

To understand the underlying mechanisms that arise from hyperplasia or dysplasia, we have carefully modified the pancreatic cancer GRN studied in [37]. The dynamics of the modified-GRN (MGRN) version reproduce well the stable states related to the proliferation and apoptosis phenotypes of PSCs and cancer cells with a good agreement in both processes. The modification of the GRN led us to introduce naturally temporal delay parameters $\tau_{1}$ and $\tau_{2}$ in the dynamics of the main regulatory genes of pancreatic cancer. The temporal delays become crucial when calibrating the clinical activation times between the different states of the PanINs’ stages. Thus, the values of $\tau_{1}$ and $\tau_{2}$ mimic the temporal progression of pancreatic cancer. From healthy to PanIN 1 onset and from PanIN 1 to PanIN 2 onset, respectively.

To investigate the convergence of the long-term temporal dynamics of the modified network, we have also analysed the network continuous logic gates [62,63]. With this latter approach, we obtained the expected gene expression profiles as an indication of the correctness of the MGRN and the approach.

**Figure 2.**
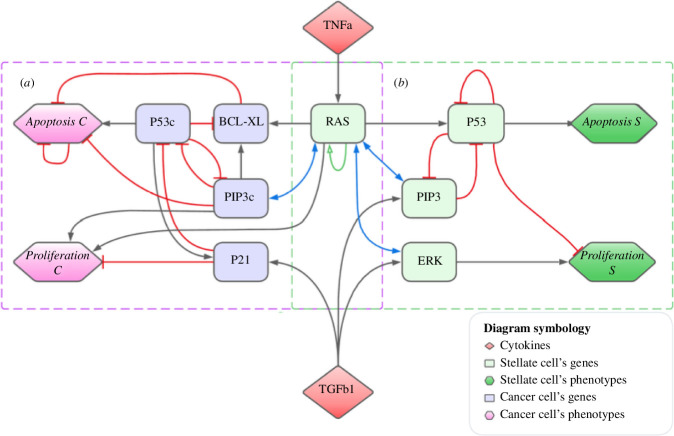
Modified GRN for a pancreas. It describes the transition from healthy cells to PanIN 1 and PanIN 2 stages. (*a*) A GRN for pancreatic cancer is represented with purple squares. (*b*) A GRN for healthy tissue is represented with green squares. The oblique lines represent inhibition actions while the arrows mean activation actions between agents. The letter ‘c’ at the end of some genes represents the action of a mutated gene.

In figure 2, we show the MGRN of pancreatic cells in which the cytokines TNFa and TGFb1 act as regulators and repressors of genes in PSCs and PCCs. Additionally, the MGRN considers the caspases (CASPs) as inflammatory response agents and apoptosis processes in cancer cells. The *RAS* gene is crucial in the MGRN since clinical data show that in all PDAC and PanIN cases, this gene is overexpressed as compared with healthy individuals. The MGRN also includes the interactions of the P53 tumour suppressor gene, the *P21* gene as well as the CDK inhibitor that acts as a checkpoint protein in the G1 cell cycle phase. The EKR extracellular kinase participates in multiple cellular processes such as proliferation, differentiation and regulation of transcription. Also, the GRN contains the *BCL-XL* gene that regulates cell death and the *PIP3* gene that causes translocation of glucose transporters to the plasma membrane and glucose uptake into tissues (for a detailed function of the agents, see table S1 in the electronic supplementary material).

**Table 1.** Optimized Boolean functions of cytokines, genes and phenotypes present in the model of the cellular microenvironment in pancreatic cancer. The variables ${\hat{y}}_{1}=y_{1}(t-\tau_{1})$ and ${\hat{c}}_{3}=c_{3}(t-\tau_{2})$ contain delay terms. For a healthy tissue, the variable $c_{3}$ in the RAS agent disappears.

agent	variable	Boolean functions	parameters
TNF α	$x_{1}$	—	n=-6
TGF β 1	$x_{2}$	—	m=-10
*RAS*	$y_{1}$	$\left(x_{1}\,||y_{3}||\,y_{4}\,||c_{3}||\,{\hat{y}}_{1}\right)$	$\epsilon_{1}=1/0.3$
*P53*	$y_{2}$	$\left(y_{1}\right)\;\&\;\left(-y_{2}\right)\;\&\;\left(-y_{3}\right)$	$\epsilon_{2}=1/2.2$
*PIP3*	$y_{3}$	$\left(y_{4}\right)\;\&\;\left(-y_{2}\right)$	$\epsilon_{3}=1$
ERK	$y_{4}$	$\left(x_{2}||y_{1}\right)$	$\epsilon_{4}=1/0.3$
*BCL-XL*	$c_{1}$	$(c_{3}||{\hat{y}}_{1})\;\&\;\left(-c_{2}\right)$	$\epsilon_{5}=1/1.25$
*P53c*	$c_{2}$	$-\left(c_{2}||c_{3}\right)\;\&\;\left(-c_{3}||c_{4}\right)$	$\epsilon_{6}=1/1.4$
*PIP3c*	$c_{3}$	$\left(-c_{2}\right)\;\&\;(\;{\hat{y}}_{1})$	$\epsilon_{7}=1$
*P21*	$c_{4}$	$\left(x_{2}||c_{2}\right)$	$\epsilon_{8}=1/0.2$
healthy apoptosis	$z_{1}$	$y_{2}$	$\epsilon_{9}=1$
healthy proliferation	$z_{2}$	$y_{3}$	$\epsilon_{10}=1$
cancer apoptosis	$z_{3}$	$\left(-c_{3})\;\&\;\left(-c_{1}||c_{2}\right)\;\&\;(z_{3}\right)$	$\epsilon_{11}=1/0.68$
cancer proliferation	$z_{4}$	$(\;{\hat{y}}_{1})\;\&\;(\;{\hat{c}}_{3})\;\&\;\left(-c_{4}\right)$	$\epsilon_{12}=$ 1/1.14

On the other hand, delayed systems of differential equations have been incorporated to mimic the delayed response of the interactions between the network components [64]. These delays are related to the biological processes of transcription, transduction and transport of molecules. The inclusion of time-delayed systems of differential equations adds more realism to the model, although introducing some difficulties when solving them. Nonetheless, it captures with more precision the temporal dynamics response of the GRN to biochemical reactions and positive feedback circuits between the network components [65].

Logic gates describe the Boolean actions of more than two variables. They regulate several input signals and result in a single output signal. Because of this, they are fundamental elements in modelling the dynamics of a GRN. In this context, the interactions between genes and proteins in a biological system can be modelled as input and output signals. Here, we use the unified Hill-function logic framework, which is an equivalence between Boolean logic and a superposition of cooperative functions [64]. In what follows, we apply this framework to model the actions of the MGRN. Let us consider a transcription factor x(t) that regulates in time the production of a protein y(t) . If x(t) activates y(t) , then the production rate of y(t) is proportional to the variations of x(t) . The cooperative interplay is modelled in the context of Hill regulation with a parameter n that determines the steepness of the increase of y(t) in response to an increase of x(t) . To account for the explicit delay in regulation, we replace x(t) with x(t−τ) . Therefore, the cooperativeness differential delay equations are


(2.1) $$\begin{array}{rl} \dot{y}(t) & =\sum_{i}\frac{\epsilon_{i}}{1+x_{i}^{n}(t-\tau )}-y(t),\;\left\{ \begin{array}{ll} -\infty <n<0 & \text{ activator}\\ n=0 & \text{ constitutive}\\ 0<n<\infty & \text{ repressor,} \end{array}\right. \end{array}$$

where the sum is over the nearest neighbours of gene y in the GRN, $\epsilon_{i}$ is the regulation strength, n is the cooperative parameter and τ is the time delay. In general, gates can exhibit a high (‘on’) or low (‘off’) output signal depending on whether the inputs are on or off. Therefore, the MGRN parameter space can be divided into two types of logic functions: AND type and OR type. The former functions are activated when both the x and y regulations are weak, while the latter functions are activated when both regulations are strong. These two types of logic functions can be interchanged at once by applying negation to one or both inputs. The set of delay differential equations that describe the dynamics of the MGRN in figure 2 are written down in electronic supplementary material (see equations (A9) in Differential equations section). Here, we write down the adimensional equations that are solved (details about the delays can be found in [57]):


(2.2) $$\begin{array}{rl} {\dot{R}}_{z}(t) & =\epsilon_{\;z}\left[\frac{1}{1+x^{n}(t-\tau_{1})}+\frac{1}{1+y^{n}(t-\tau_{2})}\right]-R_{z}(t),\\ \dot{z}(t) & =\frac{1}{1+R^{m}}-z(t). \end{array}$$

In these equations, $R_{z}$ is the regulation response of variable z due to x and y agents, and z is the regulated agent. The MGRN variables and their representation in terms of Boolean functions are presented in table 1. The activation is represented by OR and the suppression by AND NOT. Following [37], we have estimated the parameters ϵ and n of the system of delay differential equations from the attractors of the BN. In the logic scheme, we have two possible values: true and false. True and false values are represented in continuous space by ϵ greater than 1 and ϵ less than 1, respectively. Taking this into account, we chose the ϵ parameter values that map the Boolean attractors to those of the corresponding continuous dynamical system. Indeed, these attractors are related to cell phenotypes observed in the literature and clinical biopsies (see [37] and references therein).

### 2.3. Numerical integration

#### 2.3.1. Temporal analysis of the PanINs evolution

In a recent study, the average residence times at each stage of PanIN were examined [35]. It was found that the average residence time in PanIN 1 was 23.7 years, whereas the average residence time in PanIN 2 was 17.5 years. These findings indicate that the transition time from PanIN 1 to PanIN 2 is faster in cancer cells, suggesting that a temporal delay in the MGRN dynamics equations is an important point to be considered when simulating the evolution of pancreatic cancer. Because of this, here we have analysed the MGRN dynamics in terms of a set of delayed differential equations. In a comparison of the residence times in [35] between PanIN 1 onset ( $\tau_{1}$ ) and PanIN 2 onset ( $\tau_{2}$ ) for men ( m ) and women ( w ), one finds that the ratio between both times remains almost constant, that is, $\tau_{2,m}/\tau_{1,m}\approx \tau_{2,w}/\tau_{1,w}\approx 0.8$ . These ratios somehow show that the inflammatory nature in PanIN 1 is less progressive and aggressive compared with PanIN 2. From the above considerations, one can assume that cancer cells are responsible for moderating genetic deregulation by generating delays in the MGRN response with a proportion $\tau_{2}=0.8\times \tau_{1}$ . Thus, taking into account the mean values reported in [35] for the transitions between PanIN 1 onset and PanIN 2 onset, we have determined the calibration values of our model to be $\tau_{1}=17.13$ and $\tau_{2}=13.82$ years, respectively. We carried out numerical simulations using the fourth-order Runge–Kutta method to integrate the differential equations with a time step $\Delta t=10^{-4}$ that corresponds to 1/120 of the basal cell cycle. Each simulation lasted $48\times 10^{6}$ steps, equivalent to 480 months or 400 basal cell cycles. Numerical simulation details can be consulted in our GitHub repository biomodel.

#### 2.3.2. Spatial analysis of the PanIN evolution

**Figure 3.**
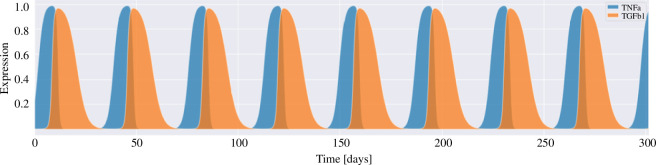
The temporal behaviour of the cytokines TNFa ( $x_{1}$ ) and TGFb1 ( $x_{2}$ ) over time. The regulated action of cytokines can be an indicator of homeostasis processes.

We have analysed the genetic–phenotypic expression profiles of PSCs in both a healthy pancreas and one with the presence of cancer cells. By considering a basal cytokine period of $T_{0}\approx 36$ days [66] and the cytokine concentrations TNFa ( $x_{1}$ ) and TGFb1 ( $x_{2}$ ) [67,68], we obtained the concentrations for cyclins E and B by using the dimensionless Lotka–Volterra equations (see equations (A4) in electronic supplementary material, where $x_{1}$ and $x_{2}$ play the role of the u and v variables, respectively). Figure 3 shows the cytokine activation cycles.

**Figure 4.**
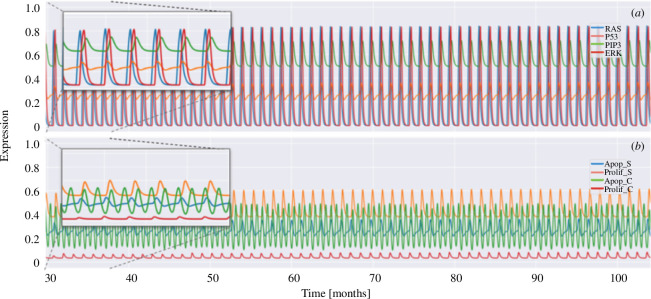
Genetic–phenotypic expression profile for genes in the MGRN of an isolated healthy pancreas. (*a*) The oscillation behaviour of the genes RAS, P53, PIP3 and ERK. (*b*) The states of cell phenotype due to proliferation (Prolif S) and apoptosis (Apop S) of healthy cells, and proliferation (Prolif C) and apoptosis (Apop C) of cancer cells. The insets show the short-time evolution of genetic agents and cell phenotypes.

Figure 4 shows the MGRN phenotypic expression results for the healthy pancreas. The MGRN exhibits a balanced and coordinated gene expression, whereas the associated phenotypes, such as proliferation and apoptosis, are at stable and controlled levels. These results indicate that the healthy pancreas is in a state of cellular homeostasis. For the evolution of a healthy pancreatic tissue, we incorporated the cytokine cycle in the MGRN since it plays a fundamental role in the genetic regulation and maintenance of homeostasis. The inset of figure 4*a* shows the short-time evolution of the genetic agents RAS, P53, PIP3 and ERK. The inset of figure 4*b* shows the healthy and cancer phenotypes, where we observe that the activity of RAS and ERK agents induces the apoptosis process periodically and maintains the proliferation of healthy cells at a high level, while the proliferation of cancer cells is inhibited.

#### 2.3.3. Spatial relaxation

To make a physical description of the acinar–ductal system inflammation process we used a Voronoi diagram construction explained in the electronic supplementary material. To this end, we have taken into account that pancreatic acinar cell injury triggers the synthesis and release of pro-inflammatory cytokines and chemokines [69–73]. To illustrate the dynamical behaviour of the system, we have considered the following parameter values in equation (A1) in the Mechanical field section in electronic supplementary material: $K_{\text{c}}=0.3$ Pa m, $K_{\text{v}}=0.06$ Pa m^−1^ as reported in [74]. These constants are related to some characteristics of healthy acinar–ductal cells such as the elastic modulus E∼ 5–10 kPa [75] and diameters of the acinar–ductal cells that vary in the interval 10–24 µm [76]. The friction parameter value in equations (A2) was k=0.001 Pa m s, and the γ=16 value in equation (A7) corresponds to one basal oscillation $T_{0}$ , when the glucose mean concentration is $\langle c_{i}\rangle =0.5$ (see Glucose transport section in the electronic supplementary material for details).

We have introduced a quantification of the inflammation in the acinar–ductal system and two key biophysical parameters: the cell size domain represented by its area $A_{\text{T}}$ and the basal cell size represented by its Voronoi area ${\hat{A}}_{\text{cell}}$ . Accordingly, the inflammation was quantified by measuring the ratio of both areas, $A_{\text{T}}/{\hat{A}}_{\text{cell}}$ . Thus, when the geometric domain occupied by the cells is under pressure as a result of inflammation, $A_{\text{T}}/{\hat{A}}_{\text{cell}}>1$ . On the contrary, when the system tissue is relaxed and inflammation is absent, cells have enough space to move around and migrate, so that $A_{\text{T}}/{\hat{A}}_{\text{cell}}<1$ . Cell proliferation increases cell density and therefore contributes to the growth of the duct in the acinar–ductal system which in turn reduces the inflammation and maintains an adequate balance in the tissue. In this situation, the following condition should be satisfied: $A_{\text{T}}/{\hat{A}}_{\text{cell}}>A_{\text{up}}$ , where $A_{\text{up}}$ is a threshold value that determines the duct elongation. In the Anti-inflammatory mechanisms section in the electronic supplementary material, we present the calculations that lead to the duct’s length.

#### Spatial cell distribution

Cell proliferation is a fundamental process in the growth and development of biological tissues. It determines the shape, density and size of the growing tissue and plays a crucial role in the formation and maintenance of the structure and function of organs. During tumour growth, the rate of cell proliferation increases so that cell division must be incorporated in the model. Taking this into account, we have followed the next four steps to emulate tissue growth: (i) identification of cells that proliferate, (ii) generation of the corresponding daughter cells, (iii) mechanical relaxation and adjustment of the tissue as a result of its growth, and (iv) cell death. Each step is described in the following paragraphs.

- Cell proliferation. According to equation (A7) of the electronic supplementary material, cells that have a cell cycle clock smaller than the average are the cells with a local glucose concentration $c_{i}$ larger than the average glucose concentration $<c_{i}>$ . Thus, the cells that proliferate more are those with an internal cell cycle clock $\left(T_{i}\right)$ smaller than the basal cell cycle clock $\left(T_{0}\right)$ . Proliferation happens when $(T_{i}-T_{0})/T_{0}>\lambda_{\text{h}}$ , where $\lambda_{\text{h}}$ is a threshold value that is chosen such that the areas of the mother and daughter cells adjust easily to the tissue’s mechanical equilibrium. We found that the threshold optimal value was $\lambda_{\text{h}}=0.15$ . This threshold was kept fixed in all numerical simulations.
- Generation of daughter cells. Cell division proceeds through precisely timed and carefully regulated stages of growth, DNA replication and division that lead to two genetically identical cells. Here, we have considered a geometrical procedure to generate the cells’ descendants. Let us assume that the shape of the two-dimensional mother cell is circular with area $A_{i}$ , so that its radius is $R_{i}=\sqrt{A_{i}/\pi }$ . In order to guarantee that the area of each daughter cell is one-half of the original mother cell area, $A_{i}$ , the centres of the daughter cells should be distant by $R_{i}/2$ from the centre of the mother cell. Then, each daughter cell will have a radius $r_{i_{k}}=R_{i}/\sqrt{2}$ , with k=1, 2 , and their initial positions are chosen randomly. In addition, we shall assume that the kinetic energy, $E_{\text{c}}$ , of the mother cell is shared with the daughter cells. After division, the masses of the daughter cells are one-half that of their mother cell, that is, $m_{1}=m/2$ and $m_{2}=m/2$ . To ensure that the daughter cells have an adequate amount of energy for their subsequent development and motility, we assume the conservation of momentum and kinetic energy during the division process. Then the magnitude of the velocity of each daughter cell becomes $\sqrt{3E_{c}/2}$ . Additionally, we assume that during cell division, the glucose concentration is shared by one-half for each daughter cell.
- Relaxation and adjustment. After cell division occurs, the tissue relaxes generating elastic interactions with the daughters’ neighbouring cells and their surroundings until an equilibrium mechanical configuration in the tissue is reached. To have synchrony between the mechanical equilibrium and the basal cell reproduction period $T_{0}$ , we shall assume that the mechanical relaxation process takes the same time $T_{0}$ .
- Cell death. Cell death is a physiological process to ensure correct development or tissue homeostasis; nonetheless, it can be considered as a pathogenic mechanism that undermines normal organ function and leads to local or systemic inflammation. Cell death can be classified into two large categories: accidental cell death and regulated cell death [77,78].

Regulated cell death plays a dual role in pancreatic cancer and has been shown to have both pro-tumourigenic and tumour-suppressive effects. At the molecular level in PDAC, various oncogenes or tumour suppressor signals determine the sensitivity of cell death modalities. The main types of regulated cell death that have been identified in PDAC are apoptosis, necroptosis, ferroptosis, pyroptosis and alkaliptosis [79]. Since the detailed mechanisms of cell death in PDAC are still to be discovered, here we simulate the cells’ death process by considering their internal cell cycle clock. To this end, we implemented the following steps. (i) Identification of candidate cells for cell death. The candidate cells for death are those that have an important difference in their internal cell cycle clock $T_{i}$ as compared the basal cell cycle clock, $T_{0}$ ; that is, when $(T_{0}-T_{i})/T_{0}>\lambda_{\text{h}}$ . When this inequality holds, the cell i is eliminated from the tissue. The previous ratio inequality is indicative that cells in latent states will die because their internal cell cycle clock is shorter than the normal one. Voronoi points associated with these cells are eliminated. (ii) Cell elimination. After cell removal, the Voronoi diagram is locally recalculated to maintain a valid and stable configuration of neighbouring cells, simulating the process of local tissue restitution.

The normal evolution of the system begins by allowing the action of mechanical and elastic fields in the tissue. Subsequently, the transport of glucose by diffusion gives rise to a gradient in the system. By solving the transport equation (A3) in the electronic supplementary material, we obtained the normalized glucose concentration. Next, the cells’ internal clock advances with increments $\Delta T_{i}=\Delta t\cdot \Delta c_{i}$ until a time of $T_{0}/2$ is reached, where Δt is the simulation time and $\Delta c_{i}$ depends on $T_{0}$ , $\gamma_{0}$ , and $\beta_{0}$ (see equation (A7) in the electronic supplementary material). When this happens, the internal cell cycle clock of each cell is checked out and a determination is made as to whether this cell either divides or dies. Once the cells’ fate is determined an iteration of equations (A2) in the electronic supplementary material is carried out in order to homogenize and stabilize the system. During this process, cells interact with each other and with their environment, adjusting their elastic equilibrium position to minimize the potential energy and reach a tissue-stationary state.

## 3. Results

### 3.1. Emergence of phenotypes from gene-regulatory network dynamics

Figure 4*a* shows that the gene *RAS* expression level remains high, whereas the gene *P53* expression level is low, as an indication of balance in stellate cell apoptosis (Apop S) and proliferation (Prolif S). Gene *ERK* shows pronounced activation values, suggesting a greater response to the growth signals. Figure 4*b* illustrates that the proliferation phenotypic expression reaches a peak as PSCs experience an increase in their proliferation rate. This is an indication that cell growth as well as renewal of the pancreatic microenvironment are underway. Alternatively, the peak in the phenotypic expression of apoptosis suggests a stable high rate of programmed cell death. This behaviour yields a bistable cycle between proliferation and death which is crucial for the dynamic balance of a healthy pancreas. In this way, healthy tissue cells respond and adapt to changes in their environment in a controlled manner, preventing the development of cellular alterations. However, a different type of dynamic behaviour in the MGRN will break the homeostatic balance and eventually lead to the development of inflammation and/or stress processes.

**Figure 5.**
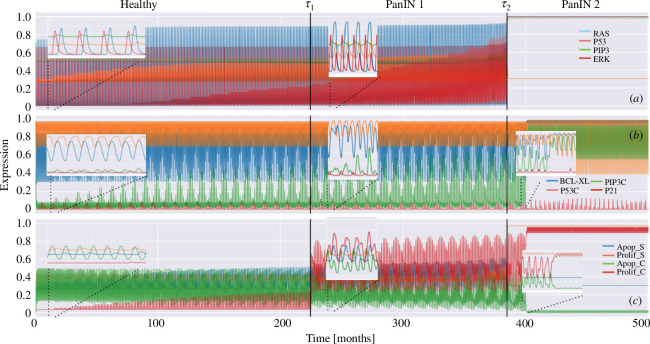
Genetic–phenotypic expression profile for the MGRN genes of the pancreas with cancer. The genes are in two states: activated or inactivated. (*a*) Oscillatory behaviour of the genes *RAS, P53, PIP3* and ERK. (*b*) Oscillatory behaviour of the cancer genes *BCL-XL*, *P53*c, *PIP3c* and *P21*. (*c*) Cell phenotype states of healthy cells due to proliferation (Prolif S) and apoptosis (Apop S), together with cell phenotype states of cancer cells due to proliferation (Prolif C) and apoptosis (Apop C). The vertical black lines correspond to the evolution of PanIN 1 and PanIN 2 stages with delay times $\tau_{1}$ and $\tau_{2}$ , respectively.

Figure 5 shows the genetic–phenotypic expression profiles for the MGRN in pancreatic cancer development. Figure 5*a,b* shows an increase in the genetic–phenotypic expression profile of the PSCs and PCCs, respectively. These results indicate that the introduction of the delayed times $\tau_{1}$ and $\tau_{2}$ in the MGRN kinetic equations is critical for the description of the pancreatic cancer progression. In fact, the delayed time $\tau_{1}$ marks the transition from the healthy pancreas to PanIN 1 stage, while $\tau_{2}$ marks the change from the PanIN 1 to PanIN 2 stage. Observe that PSC and PCC are happening approximately at the same time shortly after the beginning of the PanIN 2 stage, that is, after a time delay $\tau_{2}$ (see the inset of figure 5*c*). Also note that the regulation of *RAS*, *P53*, *PIP3* and ERK agents changes dramatically at a time delay $\tau_{2}$ .

For PanIN 1 state $\left(0<t\leq \tau_{1}\right)$ , one observes that the MGRN maintains a profile similar to that of a healthy pancreas in the PSCs. However, for the PCCs, there are some incipient alterations in some genetic agents. For instance, cancer cell proliferation (Prolif C) increases, whereas cancer cell apoptosis (Apop C) decreases, which are two cancer development hallmarks. The evolution to the PanIN 2 stage $\left(\tau_{1}<t\leq \tau_{2}\right)$ yields the activation of the ERK gene, which in turn leads to an increase in the activity of the cell growth pathway. In figure 5*c*, one observes that as time approaches the value $\left(\tau_{2}\right)$ , the proliferation of cancer cells (Prolif C) oscillates with larger amplitude and higher frequency. This suggests that cancer cells appear to be in a dormant state since they inhibit apoptosis at a slower rate. Therefore, there is a stronger and sustained activation of cancer cells, while PSCs seem to maintain a relatively stable behaviour. On the other hand, the oscillatory behaviour in the PanIN 2 stage suggests a transition to a higher proliferation rate of the PCCs, which may lead to the onset of localized tumours in the pancreas [80]. We also observe that for times less than $\tau_{2}$ the PSCs present an oscillatory behaviour that regulates their proliferation and apoptosis processes. For times greater than $\tau_{2}$ a deregulation in the processes of the PSCs is observed, suggesting an onset of a tissue inflammatory process that contributes to the proliferation of the PCCs. Furthermore, significant deregulation of the ERK gene could indirectly increase its mutations and favour the overexpression of *KRAS/RAS* as suggested in [12,37,81]. Note that this deregulation deactivates the apoptosis mechanisms in cancer cells, which favours their survival over healthy cells leading to tumour growth (see the insets in figure 5).

**Figure 6.**
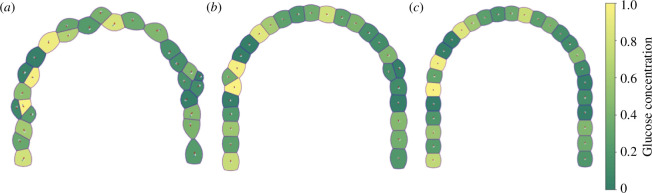
Distribution and evolution of cells subjected to their own mechanical and plastic forces without considering cell division and death. (*a*) Initial state where cells are randomly distributed. (*b*) Intermediate state. (*c*) Equilibrium state. Glucose concentration gradients are also shown and can be quantified with the colour bar located on the right-hand side.

Figure 6 shows the distribution and evolution of cells in the tissue, without considering cell division and death. They are subjected to mechanical and plastic forces such as cell adhesion and edge tension. Figure 6*a* represents an initial state where cells are randomly distributed. The mechanical field acting on cells generates deformations and changes in their shape and positions until the tissue reaches a mechanical equilibrium again (see figure 6*c*). During the relaxation process, the glucose concentration in the cells tends to distribute evenly throughout the tissue. This process facilitates GLUT diffusion across cell membranes. Notice that by construction of the Voronoi diagrams, cells are in contact leading to glucose concentration gradients in the tissue. This ensures that cells have adequate glucose supply for their metabolic processes and growth. Observe that when some cells are not in complete mechanical equilibrium steeper glucose concentration gradients may appear as shown in figure 6.

Healthy pancreas. A healthy pancreas is free of inflammation and has a balanced distribution of gene expression and cellular phenotypes. Meaning that there should be a compensation between the processes of cell division and death that maintains the homeostasis in the system. Recent findings indicate that PSCs play an important role in the healthy pancreas function, as well as in response to disease and damage. In the healthy pancreas of an adult, PSCs are present in small amounts, nonetheless, they are ‘quiescent’ and regulate extracellular matrix (ECM) production. On the other hand, activated PSCs proliferate rapidly and undergo substantial changes in gene expression. They are perhaps the most abundant cell type of a damaged pancreas. Because of this, PSCs have been extensively studied to understand their role in pancreatitis and cancer [82–85].

### 3.2. PanIN 1 (low-grade dysplasia)

**Figure 7.**
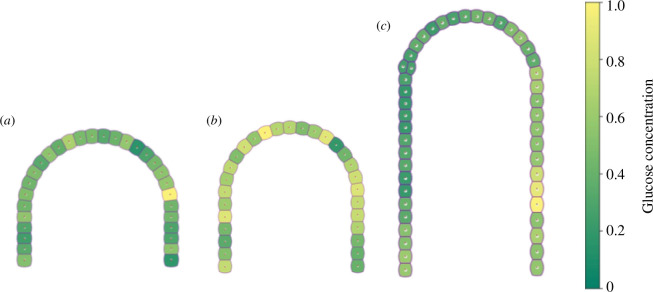
Evolution of an acinar–ductal system: (*a*) healthy, (*b*) soft inflammation and (*c*) moderate inflammation. Glucose gradient concentration is indicated according to the colour bar on the right-hand side.

Pancreatic cancer precursor lesions at PanIN 1 stage are characterized by the onset of an inflammation process during which healthy cells are transformed into precancerous cells. Because of this, we have considered different inflammation scenarios based on gene expression and cell proliferation to mimic the effects of inflammation. The acinar–ductal system evolution through the PanIN 1 stage starting from a healthy state is shown in figure 7. Figure 7*a* shows a healthy acinar–ductal system followed by light and moderate inflammation states that are shown in figure 7*b,c*, respectively. One observes an increase of cell proliferation and the cellular area/volume ratio leading to inflammation. A typical process that characterizes the PanIN 1 stage is that during duct elongation, the increase in the cell area/volume contributes to relieve stress on pancreatic cells. Figure 7 shows that during the inflammation, the glucose diffuses through some regions of the acinar–ductal system. We observed that there are regions where glucose concentration diminishes significantly pointing to high consumption of glucose indicating changes in cell metabolism as a result of inflammation.

### 3.3. PanIN 2 (moderate-grade dysplasia)

PanIN 2 lesions appear to be columnar and contain nuclear changes within the cells such as the loss of nuclear polarity, nuclear crowding, different nuclear sizes, nuclear hyperchromasia and nuclear pseudostratification [86,87]. During the progression from PanIN 1 to PanIN 2, mutations in the CDKN2A gene occur frequently which are associated with the loss of p16 expression [88]. As a result, a progressive increase in cell proliferation and survival of cancer cells is observed during the transition from normal ducts to PDAC [89]. Because of this, in the present qualitative model, at least one cancer cell has been added to the cellular dynamics in the evolution from PanIN 1 to PanIN 2. These cells are able to proliferate since they are no longer in a quiescent state.

**Figure 8.**
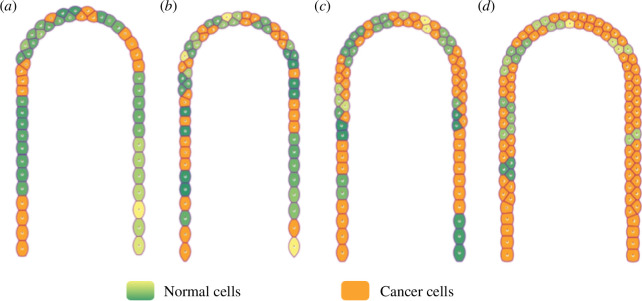
Four-year cancer evolution structures initiated with (*a*) six, (*b*) ten, (*c*) fourteen and (*d*) eighteen cancer cells. It is observed that inflammation evolves from moderate in (*a,b*) to severe in (*c,d*). The scale of the colour of normal cells represents glucose concentration as is indicated in the colour bar in figure 7.

**Figure 9.**
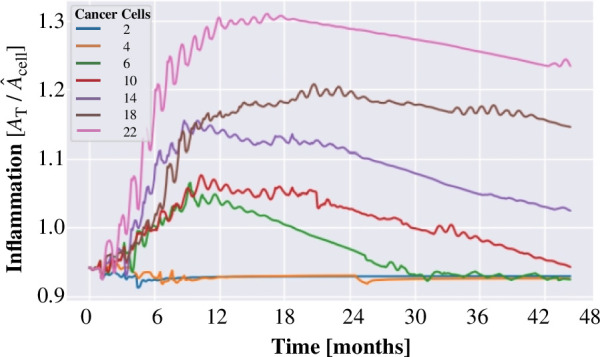
Evolution of the pancreas inflammatory process for different initial amounts of cancer cells.

To understand how the density of cancer cells affects the proliferative activity of the system as well as the rate of progression of PanIN 2 lesions, we have analysed the response of the system to the inclusion of cancer cells in four representative biological scenarios. We show 4-year-old cancer evolution structures in figure 8*a*–*d* initiated with six, ten, fourteen and eighteen cancer cells, respectively. The structure presented in figure 8*a* suggests that the six initial cancer cells remain in a quiescent state because the proliferation rate is negligible. Figure 8*b* shows a structure with 10 initial cancer cells where cell proliferation originates at different places indicating that cancer cells are no longer in a quiescent state. Figure 8*c* shows a structure that originated with 14 cells. In this case, we observe that after 4 years, a moderate density of cells triggers a significant tumour growth rate. In fact, we can realize that there occurs a local formation of small tumours. The structure shown in figure 8*d* was initiated with 18 cancer cells. After 4 years of progression, the high proliferation triggers the tumour growth rate. The inflammation index increases to severe levels and drives the transition to PanIN 3. In this stage, there should be a greater degree of aggressiveness as compared with the structures shown in figure 8*a*,*b*. As expected, these results indicate that as the initial density of cancer cells increases there will form more localized tumours with greater density that eventually yield a big dense tumour. Figure 9 shows the evolution of inflammatory process for different initial amounts of cancer cells as a measure of the pancreas homeostatic state. We observe that for a small initial amount of cancer cells, the pancreas system eventually recovers its equilibrium state. However, as the initial amount of cancer cells increases, it is shown that the pancreas never returns to its homeostatic state. Thus, if all cancer cells were removed, the pancreas would recover its equilibrium state.

### 3.4. PanIN 3 (high-grade dysplasia)

These lesions rarely are flat, their structure is micropapillary or papillary and are the result of neoplastic epithelial cell proliferation. They form large nests perforated by many rounded different-sized spaces that eventually give rise to small clusters of epithelial cells in the lumen. Cytologically, these lesions are characterized by a loss of nuclear polarity, dystrophic goblet cells (goblet cells with nuclei oriented towards the lumen and mucinous cytoplasm oriented toward the basement membrane), mitoses that may occasionally be abnormal, nuclear irregularities, and prominent (macro) nucleoli. The lesions resemble carcinoma at the cytonuclear level, but invasion through the basement membrane is absent. PanIN 3 lesions have a complex structure with a papillary morphology and enlarged nuclei [90,91]. They may form clusters of cells that are removed from the epithelium into the lumen of the duct [87]. In addition, it has been found that many cellular signals and pathways contribute to the activation of PSCs, for instance, TGF- β , platelet-derived growth factor (PDGF), MAPK, Smads, nuclear factor κB , to mention only a few [92].

## 4. Discussion and conclusions

The model presented and analysed here can mimic a healthy pancreas with a homeostatic state and a balanced cellular dynamics. The cells in the organ proliferate in a controlled manner and respond to programmed apoptosis in an orderly and sequential manner. Furthermore, the model showed that no significant changes occur in the cellular structure at the macroscopic level and that glucose concentrations remain relatively stable. The latter suggests that relaxation of the system is a process in which cells adjust their cell cycle to synchronize it with their neighbours and return to equilibrium. This cellular synchronization could be considered as a form of cellular cooperation, where cells work together as a whole to maintain a healthy state and prevent uncontrolled proliferation. This process occurs through a complex network of interactions and factors so that when there happens a failure, it may affect its coherence and stability. For instance, a network failure may lead to stress and inflammation of the tissue which may perturb glucose sources, lead to hypoxia among other factors.

During the growth of the acinus in response to inflammation in the early PanIN 1 stages, the model showed that elongation of the acinar duct and changes in the spatial distribution of cells lead to a stress reduction. Furthermore, to cope with the stress caused by inflammation, an increase in cell proliferation and growth of the acinus structure was observed. We can consider this phenomenon as a resilience effect of the system in PanIN 1 since the acinar–ductal system has the capacity to recover from an inflammatory state and return to a state without inflammation (see figure 7). Likewise, when the system is allowed to relax and undergo slight growth in the acinus (especially in the case of moderate inflammation), the pancreas can return to its stable, healthy state without resorting to cell proliferation.

The presence of a small number of active cancer cells due to the accumulation of genetic alterations (PanIN 2) is sufficient for tumour formation (see figure 8). We observed that the proliferation rate of cancer cells increases as the proportion of these malignant cells grows in the system. Additionally, cancer cells compete for available space to grow as local tumours while healthy cells in the pancreas act as barriers that delineate one tumour from another. The model shows that the progression of cancer cells is sensitive to the initial proportion of them in the system (see figure 8). When a greater number of cancer cells are present in the PanIN 1 and PanIN 2 stages, the rate of progression of PanINs to more advanced cancer stages increases as the results of figure 8*d* suggest. This is interesting as it indicates that controlling cancer cells in early stages (PanIN 1) can prevent the rapid progression of pancreatic cancer. A significant increase in the proportion of cancer cells in the pancreas is an indication of the transition to PanIN 3. In this advanced stage, inflammation increases to severe levels suggesting the possibility of tumour hypoxia and, if boundaries are breached, a greater potential for invasion throughout the pancreas acinar–ductal system develops. It should be noted that spatial dynamics shows emergent behaviour as cancer cells interact and compete for the available space to proliferate (see figure 8*c,d*). This cellular competition has important implications in the formation and expansion of tumours that proliferate depending on the aggressiveness and proportion of cancer cells. Healthy cells act jointly and synchronized to slow the growth of cancer by acting as natural barriers.

On the other hand, gene expression profiles show a snapshot of the transformation from a healthy pancreas to a pancreas with cancer, revealing the importance of changes in gene expression during the progression of this disease. Our findings presented in figure 5*a* underline the relevance of several genes in the MGRN that undergo notable alterations throughout different stages of pancreatic cancer progression. In particular, we have identified *KRAS/RAS* as a critical player in the initiation and growth of pancreatic cancer. The deregulated activation of this gene that drives cancer cell proliferation emerges as an essential starting point in the early stages of the disease. Furthermore, variation in *P53* expression sheds light on its contribution to the evasion of cellular controls and helps achieve cancer cells’ survival at later stages. In figure 5*b*, deregulation of *PIP3* at early stages, especially in the PanIN 1 stage, offers important clues about its involvement in the inhibition of pro-apoptotic signals, which promotes cell survival and facilitates the transition to more aggressive states. The potential to activate the *RAS* pathway at the PanIN 2 stage is related to ERK overexpression which promotes cell proliferation and malignant transformation.

Activation of PIP3 and gradual inactivation of CASP in early stages may facilitate more effective cell survival and proliferation, paving the way to the transition to the more aggressive PanIN 3 stage. At the PanIN 3 stage, overexpression of *RAS*, together with permanent inactivation of CASP, can trigger evasion of cellular control and apoptosis, driving tumour growth and cancer progression. These discoveries not only enrich our understanding of pancreatic cancer progression but also present exciting opportunities for early diagnosis and effective treatment. Prominent genes such as *KRAS/RAS*, *P53*, *PIP3* and ERK emerge as potentially useful biomarkers for monitoring the disease evolution, given their correlation with the phenotypic changes observed at the different stages.

The proposed model has the potential to be extended by considering the role of the immune system in the transformation of healthy cells to cancer cells through a better comprehension of the regulatory networks related to inflammatory processes. The main goal of this paper has been to show that the spatio-temporal evolution from healthy cells to cancer cells in the pancreas has its bases in the inflammatory process. Therefore, a better understanding of this dynamics may improve clinical practice by providing valuable information for early diagnosis as well as the development of personalized therapies.

## Data Availability

Mathematical concepts and formulations are reviewed in the electronic supplementary material. Data and relevant code for this research work are publicly available on GitHub: joshbrx/biomath_model and have been archived within the Zenodo repository [93]. Supplementary material is available online [94].
